# Requirement for sphingosine kinase 1 in mediating phase 1 of the hypotensive response to anandamide in the anaesthetised mouse

**DOI:** 10.1016/j.ejphar.2018.10.027

**Published:** 2019-01-05

**Authors:** Fiona H. Greig, Katrin Nather, Margaret D. Ballantyne, Zeshan H. Kazi, Husam Alganga, Marie-Ann Ewart, Karolina E. Zaborska, Bracy Fertig, Nigel J. Pyne, Susan Pyne, Simon Kennedy

**Affiliations:** a Institute of Cardiovascular and Medical Sciences, College of Medical, Veterinary & Life Sciences, University of Glasgow, G12 8QQ, UK; b Cell Biology Group, Strathclyde Institute of Pharmacy and Biomedical Science, 161 Cathedral Street, University of Strathclyde, Glasgow G4 0RE, UK

**Keywords:** Sphingosine kinase, Sphingosine-1-phosphate, Hypotension, Anandamide, Mouse

## Abstract

In the isolated rat carotid artery, the endocannabinoid anandamide induces endothelium-dependent relaxation *via* activation of the enzyme sphingosine kinase (SK). This generates sphingosine-1-phosphate (S1P) which can be released from the cell and activates S1P receptors on the endothelium. In anaesthetised mice, anandamide has a well-characterised triphasic effect on blood pressure but the contribution of SK and S1P receptors in mediating changes in blood pressure has never been studied. Therefore, we assessed this in the current study.

The peak hypotensive response to 1 and 10 mg/kg anandamide was measured in control C57BL/6 mice and in mice pretreated with selective inhibitors of SK1 (BML-258, also known as SK1-I) or SK2 ((R)-FTY720 methylether (ROMe), a dual SK1/2 inhibitor (SKi) or an S1P_1_ receptor antagonist (W146). Vasodilator responses to S1P were also studied in isolated mouse aortic rings.

The hypotensive response to anandamide was significantly attenuated by BML-258 but not by ROMe. Antagonising S1P_1_ receptors with W146 completely blocked the fall in systolic but not diastolic blood pressure in response to anandamide. S1P induced vasodilation in denuded aortic rings was blocked by W146 but caused no vasodilation in endothelium-intact rings.

This study provides evidence that the SK1/S1P regulatory-axis is necessary for the rapid hypotension induced by anandamide. Generation of S1P in response to anandamide likely activates S1P_1_ to reduce total peripheral resistance and lower mean arterial pressure. These findings have important implications in our understanding of the hypotensive and cardiovascular actions of cannabinoids.

## 1 Introduction

In a previous study (Mair et al., 2010), we identified a novel pathway which may underlie the endothelium-dependent vasodilator effects of anandamide in the rat coronary artery. This pathway involves the activation of sphingosine kinase 1 (SK1), the enzyme that catalyses the phosphorylation of sphingosine to produce sphingosine-1-phosphate (S1P), release of S1P and activation of S1P receptors on the vascular endothelium. Thus we proposed that this pathway allows S1P to access local populations of S1P receptors (Mair et al., 2010). Since anandamide has well-documented effects on blood pressure (BP), we sought to investigate if SK is required for anandamide to induce hypotension in anaesthetised mice and if S1P receptors are involved.

Anandamide (AEA) is an endogenously-generated cannabinoid which activates the endocannabinoid receptors CB_1_ and CB_2_ and the vanilloid transient receptor potential channels of V1 type (TRPV1) receptor (Stanley and O'Sullivan, 2014). These endocannabinoids might also activate as yet uncharacterised receptors within the cardiovascular system, such as the orphan G protein-coupled receptors (GPCRs), GPR55 (Ryberg et al., 2007, Johns et al., 2007) and GPR119 (Overton et al., 2006). Both GPR55 and GPR119 have been demonstrated to bind endocannabinoids and evidence also exists for a non-CB_1_/non-CB_2_ endothelial receptor, CB_x_ (Offertaler et al., 2003, Zakrzeska et al., 2010) although this is controversial and requires further investigation. Both CB_1_ and CB_2_ receptors are located within the cardiovascular system (Batkai et al., 2004) and CB_2_ receptors are also present on circulating immune cells (Munro et al., 1993). The cardiovascular actions of endocannabinoids are complex and vary not only between species but also with experimental conditions (Randall et al., 2004). In anaesthetised animals, intravenous (i.v.) administration of AEA evokes a triphasic response (Lake et al., 1997, Malinowska et al., 2001, Pacher et al., 2004). The initial phase I is characterised by a rapid and transient drop in BP which is accompanied by bradycardia, increased total peripheral resistance (TPR) and reduced cardiac contractility. Phase II consists of a short pressor response, marked by an increase in BP, cardiac contractility and mesenteric blood flow. This is followed by a more prolonged (~10 min) hypotension, a reduction in mesenteric blood flow, cardiac contractility and heart rate (HR) (Malinowska et al., 2012). The hypotensive phase I response is thought to involve a vagal-mediated Bezold-Jarisch reflex and the activation of TRPV1 (Malinowska et al., 2001, Pacher et al., 2004). TRPV1 is expressed widely in the cardiovascular system, including on blood vessels (Zhang et al., 2015, Fernandes et al., 2012) and so AEA, or mechanisms downstream could affect blood vessels as well as the heart during phase I. However, to date no study has addressed whether the sphingolipid pathway is involved in mediating the phase I hypotensive response to AEA. Based on our *in vitro* data in the rat coronary artery (Mair et al., 2010), we hypothesised that generation of S1P in response to i.v. administration of AEA may underlie the phase I hypotensive response in the mouse.

S1P is a lysophospholipid derived from phosphorylation of sphingosine. S1P can function inside cells to bind to target proteins such as histone deacetylase 1/2 (reviewed in Pyne and Pyne, 2011). Extracellular S1P can also bind to high affinity GPCRs (S1P_1–5_), of which S1P_1,_ S1P_2_ and S1P_3_ are localised within the cardiovascular system (Pyne and Pyne, 2011). S1P mainly functions as a pro-survival signalling molecule while sphingosine is associated with pro-apoptotic pathways and is an important regulator of cell stress responses (Hannun and Obeid, 2002). SK catalyses the formation of S1P from sphingosine and hence represents a key checkpoint in the regulation of the relative levels of sphingosine and its precursor, ceramide, and S1P; termed the sphingolipid rheostat. Two distinct SK isoforms have been identified called SK1 and SK2 (Kohama et al., 1998, Liu et al., 2000). The two isoforms differ substantially in their tissue expression, substrate and inhibitor specificity, kinetic properties as well as their cellular localisation (Chan and Pitson, 2013). SK1 is predominately localised in the cytoplasm of cells (Kohama et al., 1998, Olivera et al., 1998). In response to agonist-stimulation, SK1 is phosphorylated, activated several-fold and translocated to the plasma membrane (Pitson et al., 2003). In contrast, phosphorylation of a nuclear export sequence in SK2 promotes its export from the nucleus (Ding et al., 2007).

SK/S1P has been implicated in negatively regulating BP in hypertension (Spijkers et al., 2012) and growing evidence suggests a link between the sphingolipid and endocannabinoid signalling systems. Phylogenetic analysis has identified a ~20% sequence homology between S1P and CB receptors and CB_1_ activation was shown to activate enzymes involved in sphingolipid metabolism (Galve-Roperh et al., 2000, Gustafsson et al., 2009). Furthermore, we (Mair et al., 2010) and others have presented evidence to suggest that S1P can act as an agonist at CB receptors and that the vascular effects of AEA require SK1 (Paugh et al., 2006). Therefore, the aim of this study was to identify the contribution of the two SK isoforms to the phase I hypotensive action of AEA *in vivo*. We also examined whether S1P released from cells as a consequence of the activation of SK uses S1P receptors to induce this hypotensive response.

## 2 Materials and methods

### 2.1 In vivo experiments

All animal care and experimental procedures were in accordance with the UK Animals (Scientific Procedures) Act 1986 and data are reported according to the ARRIVE (Animal Research: Reporting of *In Vivo* Experiments) guidelines. Ethical approval was granted by the University Ethics Committee and conformed to institutional regulations at the University of Glasgow. All mice used in the study were bred in the University of Glasgow, kept on a 12 h light/dark cycle and fed *ad libitum*.

### 2.2 Administration of drugs

Twenty four hours prior to BP measurements, animals were randomly assigned to receive one of the following treatments *via* intraperitoneal (i.p.) injection: 75 mg/kg of the dual SK1/2 inhibitor, 2-(*p*-hydroxyanilino)-4-(*p*-chlorophenyl) thiazole (SKi, Calbiochem, San Diego, CA, U.S.A. (French et al., 2003)); 75 mg/kg of the selective SK1 inhibitor, (2 *R*,3 *S*,4*E*)-*N*-methyl-5-(4′-pentylphenyl)-2-aminopent-4-ene-1,3-diol (BML-258 (also known as SK1-I), Tocris Bioscience, Bristol, U.K. (Paugh et al., 2008)); 75 mg/kg of the selective SK2 inhibitor, (*R*)-FTY720 methyl ether (ROMe) (Lim et al., 2011) or the equivalent volume of vehicle (0.1 ml of dimethyl sulphoxide (DMSO)). To test the effect of blocking S1P_1_ on the hypotensive response to anandamide, we employed 10 mg/kg of the selective antagonist, (*R*)-3-amino-4-(3-hexylphenylamino)-4-oxobutylphosphonic acid trifluoroacetate (W146, Avanti Polar Lipids, Alabaster, U.S.A. (Tarrason et al., 2011)), which was injected 30 min prior to BP measurement. Control animals received an equivalent volume of solvent (0.1 ml DMSO). All doses of drugs used were based on previously published studies.

### 2.3 BP recording

Haemodynamic measurements were performed under inhalational anaesthesia. Male C57BL/6 mice (mean weight 24 ± 3.5 g) were induced using 3% isoflurane supplemented with oxygen and maintained on 1.5% isoflurane in oxygen throughout the procedure. The left carotid artery was exposed and cannulated with a heparinised saline-containing cannula (Harvard Apparatus, Kent, U.K.) connected to a Bio-Pac Student Lab Pro pressure transducer and recorder (BioPac Systems, Norfolk, U.K.). The right jugular vein was cannulated for administration of AEA (1 or 10 mg/kg in tocrisolve), methanandamide (1 or 10 mg/kg in tocrisolve) or the equivalent volume of tocrisolve (0.1 ml per injection). In all animals, BP was recorded continuously and 5 min was allowed between injections of AEA, methanandamide or vehicle at which time BP had stabilised. All injections were given rapidly over 1–2 s.

### 2.4 Wire myography

After mice (mean weight 33 ± 1.8 g) were killed with a rising concentration of CO_2_, the aorta was removed and transferred to oxygenated modified Krebs-Henseleit (KH) solution containing (in mM): 118 NaCl, 25 NaHCO_3_, 4.7 KCl, 1.2 KH_2_PO_4_, 1 MgSO_4_, 2.5 CaCl_2_, 11 glucose. The aorta was used in these experiments as, although it is not a resistance vessel, the functional responses and S1P receptor populations have recently been reported (Roviezzo et al., 2014). The aorta was carefully dissected to remove surrounding connective tissue and, in some experiments, the lumen was rubbed gently to remove endothelium and mounted in a wire myograph containing KH solution at 37 °C and aerated with 95% O_2_/5% CO_2_. After a period of equilibration, the vessels were placed under an optimum resting tension of 1 g for at least 30 min then challenged with sequential addition of 40 and 80 mM KCl to assess viability. In experiments with denuded vessels, successful removal of the endothelium was verified by contracting with 30 nM of the thromboxane A_2_ analogue, U46619 and adding 10 µM acetylcholine. Lack of relaxation in response to acetylcholine indicated no endothelium was present.

To assess the effects of increasing concentrations of sphingosine, S1P and AEA on aortic vascular tone, vessels were pre-contracted with 30 nM U46619. A dose-response curve was generated by the cumulative addition of AEA (1 nM to 100 µM), sphingosine and S1P (1 nM to 5 µM) in half-log molar concentrations. In some experiments, the effect of the dual SK1/2 inhibitor SKi, on agonist-induced relaxation was investigated. In these experiments, 10 µM SKi was added 5 min prior to the addition of U46619 and was present throughout the generation of the dose-response curve. To investigate the receptor subtypes responsible for the S1P-mediated vasodilatation in the mouse aorta, rings were pre-incubated for 10 min with either 10 µM W146 or 10 µM of the combined S1P_1/3_ antagonist, 2-amino-*N*-(3-octylphenyl)-3-(phosphonooxy)-propanamide (VPC 23019, Tocris Bioscience, Bristol, U.K. (Davis et al., 2005)). Inhibitors remained in the bath for the duration of the experiment. For all experiments, sphingosine and S1P were prepared and diluted in a 4 mg/ml aqueous solution of bovine serum albumin (BSA) and stored on ice (Rosen et al., 2009).

### 2.5 Immunohistochemistry

For analysis of S1P_1_ expression and location, aortae from vehicle- and W146-treated mice were carefully excised following cervical dislocation. The aortae were cleared of any surrounding fat and connective tissue and fixed in 10% formalin at 4 °C for 24 h. Following fixation, the aortae were dehydrated, paraffin-embedded and sectioned on a rotary microtome at 5 µm. Slides were subjected to a standard immunohistochemical protocol to study S1P_1_ expression (Cuevas et al., 1994). Briefly, antigens were retrieved by microwave pressure cooking in citric acid followed by blocking of endogenous peroxidase and non-specific binding. Primary antibody against S1P_1_ was then incubated overnight at 4 °C (1:150 dilution in 1% (w/v) BSA in PBS, Abcam, Cambridge, U.K.). A peroxidase-labelled DAB method followed by counterstaining using haematoxylin was used to visualise the location of S1P_1_ on the aortae.

### 2.6 Statistical analysis

Data are expressed as mean ± S.E.M. where *n* is the number of different animals or number of aortae from individual animals. All statistical analyses were performed using GraphPad Prism 5.0 (La Jolla, CA, U.S.A.). Differences in baseline mean arterial blood pressure (MAP) were analysed by either unpaired *t*-test or one-way ANOVA with Dunnett's post hoc test. The percentage change in MAP and HR in response to anandamide ± SK inhibitors or S1P receptor antagonists were analysed by two-way ANOVA with Bonferroni's post-hoc analysis. For myography experiments, comparisons of drug incubation on U46619 contraction were analysed by either unpaired Student's *t*-test or one-way ANOVA while dose-response curves were analysed using two-way ANOVA. P < 0.05 was considered statistically significant.

## 3 Results

### 3.1 Effects of SK inhibitors

**Fig. 1:**
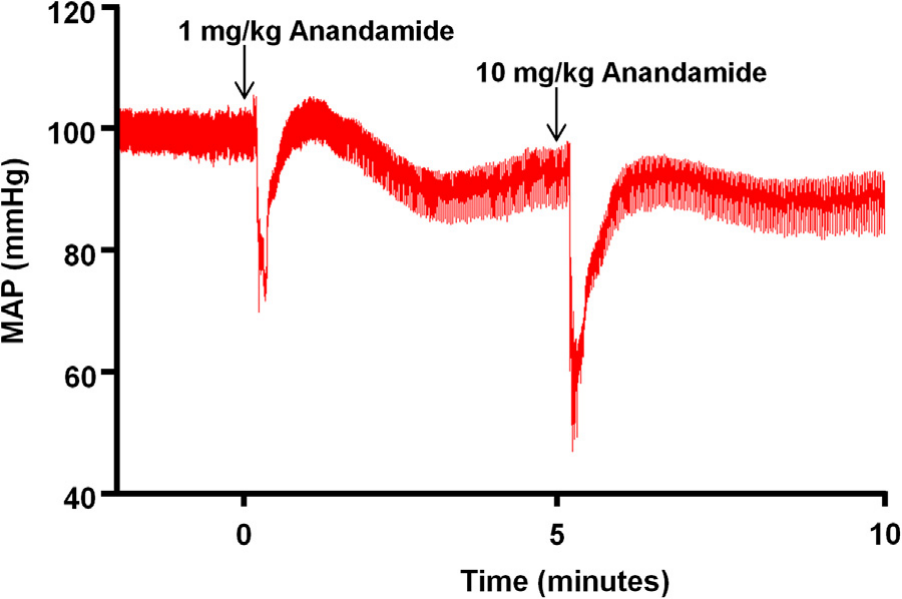
Representative experimental recording showing the changes in BP induced by i.v. injection of two doses of anandamide (1 and 10 mg/kg) in mice. Arrows indicate the injections of anandamide which were administered at 5 min intervals.

**Fig. 2:**
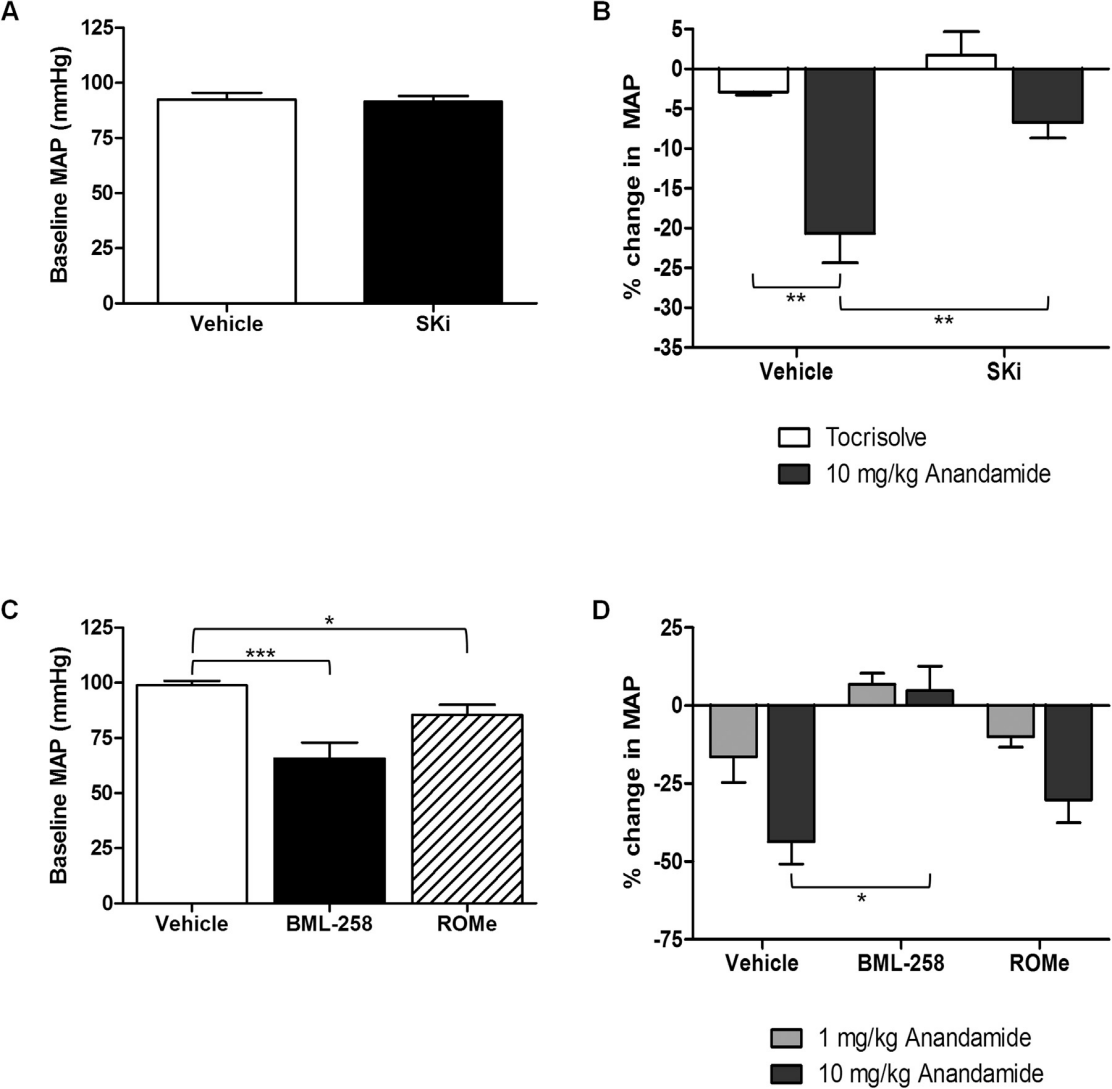
Effect of pretreatment with SK inhibitors on the peak hypotensive response to i.v. anandamide administration. (A) Mice were pre-treated with either vehicle or dual SK1/2 inhibitor, SKi (75 mg/kg) for 24 h prior to baseline MAP measurement. n = 12. (B) Baseline values were compared to the peak hypotensive response following tocrisolve or anandamide injection. **P < 0.01, n = 4–8, two-way ANOVA. (C) Mice were pre-treated with either vehicle or the selective SK1 or SK2 inhibitors (75 mg/kg), BML-258 or ROMe respectively, for 24 h prior to baseline MAP measurement. *P < 0.05 and ***P < 0.001, n = 5–12, one-way ANOVA. (D) Baseline values were compared to the peak hypotensive response following increasing doses of anandamide. *P < 0.05, n = 5–9, two-way ANOVA.

**Fig. S1:**
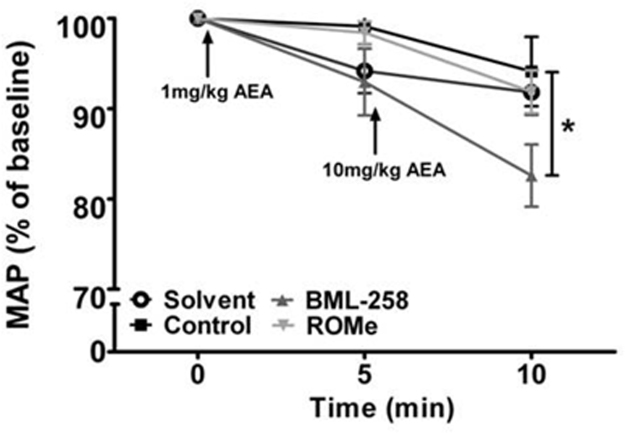
Recovery time of MAP following injection of AEA. Mice were pre-treated with either DMSO (control; n = 9), BML-258 (n = 5) or ROMe (n = 4) 24 h prior to the i.v. injection of AEA or Tocrisolve (solvent; n = 6). AEA was injected at increasing doses of 1 and 10 mg/kg at 5 min intervals. To assess the duration of the phase 3 response induced by AEA and the time taken to return to baseline, MAP was analysed 5 min after the injection of the relevant dose and expressed as percentage of baseline MAP. In all groups, MAP had recovered to baseline levels 5 min after injection of the lower dose of AEA. Data are expressed as mean ± S.E.M. *P < 0.05 *vs.* control.

AEA induced a dose-dependent, transient fall in BP, followed by a short pressor response and a longer lasting hypotension (Fig. 1). In all experimental groups, MAP had recovered to a level not significantly different from pre-injection levels 5 min after the 1 mg/kg dose of AEA (Supplementary Fig. 1). Thus, the initial dose of AEA is unlikely to have influenced the response to the second injection of 10 mg/kg AEA. Baseline mean arterial blood pressure (MAP) was 92.6 ± 3.0 mmHg in vehicle-treated mice (n = 12) which was not significantly different from animals injected with the dual SK1/2 inhibitor, SKi (75 mg/kg) 24 h previously (91.6 ± 2.6 mmHg; n = 12, Fig., 2A). However, baseline HR was reduced by SKi treatment (495.4 ± 22.4 bpm in control; n = 12 *versus* 421.3 ± 22.3 bpm in SKi group; n = 12). Since baseline blood pressure data were very consistent within experimental groups, reductions are reported as % values. In mice treated with SKi, the hypotensive response to AEA was inhibited (20.7 ± 3.7% of baseline with vehicle (n = 8) *versus* 6.7 ± 1.9% of baseline with SKi (n = 7), Fig. 2B). AEA also had a tendency to lower HR during phase I in control and SKi-treated animals although this was not significant (455.0 ± 40.8 bpm in DMSO-treated animals *versus* 412.0 ± 28.5 bpm in SKi-treated animals following AEA administration; n = 5–8). Methanandamide (10 mg/kg) also caused a rapid phase I hypotensive response but this was smaller compared to AEA (9.9 ± 6.6% of baseline value; n = 3). Tocrisolve, the AEA vehicle, had no effect on MAP (Fig. 2B) or on HR (data not shown).

**Fig. S2:**
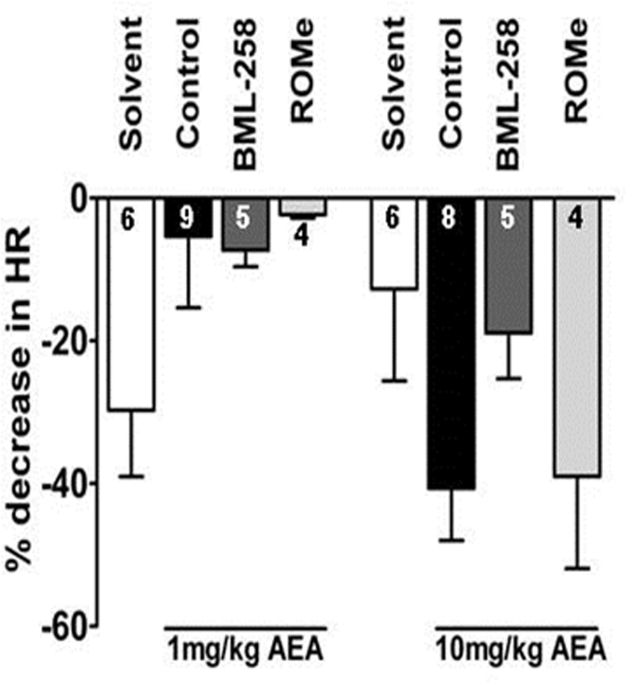
Variable bradycardic response during phase 1 hypotension. Mice were pre-treated with either DMSO, (control) BML-258 or ROMe (both 75 mg/kg) and anaesthetised with isoflurane. The %-decrease from pre-injection values for HR during the initial hypotension in response to 1 and 10 mg/ml AEA or the equivalent amount of tocrisolve solvent was quantified for the different treatment groups. Data are expressed as mean ± S.E.M. Numbers in the bars denote *n*-numbers. P = ns for comparisons made with the control group.

In order to determine which SK isoform was important in mediating the AEA-induced hypotension, these experiments were repeated using SK1- and SK2-selective inhibitors, BML-258 (SK1-I) and ROMe, respectively. Neither inhibitor had any effect on baseline HR but ROMe and, to a greater extent, BML-258 significantly lowered baseline MAP (Fig. 2C). In mice treated with the vehicle, the maximum phase I hypotensive response to AEA was 16.5 ± 8.2% of baseline MAP at 1 mg/kg (n = 9) and 43.6 ± 7.2% of baseline MAP at 10 mg/kg (n = 9; Fig. 2D). In mice pretreated with BML-258, the hypotension induced by AEA was abolished and instead a small increase in MAP was observed (6.8 ± 3.6% at 1 mg/kg (n = 5) and 4.8 ± 7.8% at 10 mg/kg (n = 5)). Conversely, ROMe had no significant effect on the hypotension induced by AEA (Fig. 2D). As found previously, AEA caused some transient bradycardia but there were no significant differences in the magnitude of the bradycardia across the groups at either dose of AEA (Supplementary Fig. 2). Tocrisolve treatment had no effect on either MAP or HR in either vehicle-, BML-258- or ROMe-pretreated animals.

### 3.2 Effect of an S1P_1_ antagonist

**Fig. 3:**
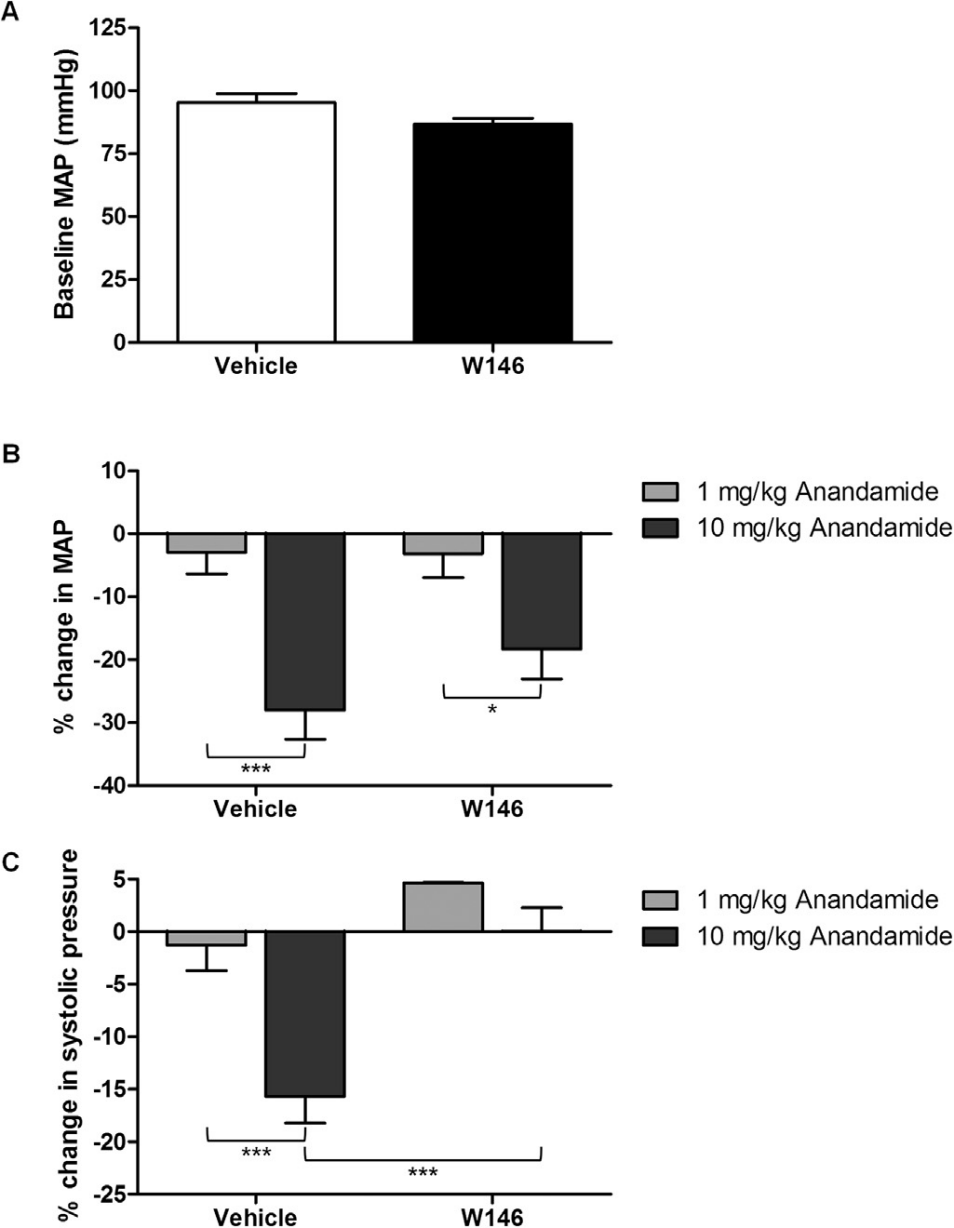
Effect of the S1P_1_ antagonist W146 on the peak hypotensive response to i.v. administration of anandamide. (A) Mice were pre-treated with either vehicle or W146 (10 mg/kg) for 30 min prior to baseline MAP measurements. n = 6–12. (B and C) Baseline MAP and systolic BP values were compared to the peak hypotensive response following increasing doses of anandamide. *P < 0.05 and ***P < 0.001, n = 5–6, two-way ANOVA.

Our data suggest that injection of AEA causes the generation of S1P catalysed by SK1 which is responsible for the phase I hypotensive effect. We therefore hypothesised that the vascular component of the hypotensive response may be mediated *via* activation of S1P_1_ on the vasculature. To test this, we pretreated mice with the selective S1P_1_ antagonist W146 (10 mg/kg) for 30 min before recording the responses to AEA. W146 treatment did not significantly affect baseline MAP (Fig. 3A) but attenuated the hypotensive response to 10 mg/kg AEA (Fig. 3B). Tocrisolve had no effect on MAP or HR in either the vehicle- or W146-treated mice (data not shown). Interestingly, when systolic and diastolic pressure changes were analysed, it could be seen that W146 completely attenuated the fall in systolic pressure induced by 10 mg/kg AEA (-15.7 ± 2.5 mmHg in vehicle-treated mice (n = 8) *versus* 0.04 ± 2.3 mmHg in W146-treated mice (n = 8); Fig. 3C) but had no effect on the diastolic pressure (data not shown). Thus, mice treated with W146 experience a significant increase in pulse pressure following 10 mg/kg AEA injection (36.6 ± 6.3 mmHg with vehicle (n = 5) *versus* 55.0 ± 2.2 mmHg with W146 (n = 5)). Again, AEA-induced a bradycardic effect which was not significantly different in W146 *versus* vehicle-treated mice (data not shown).

### 3.3 Vasodilation to S1P in isolated mouse aortae

**Fig. 4:**
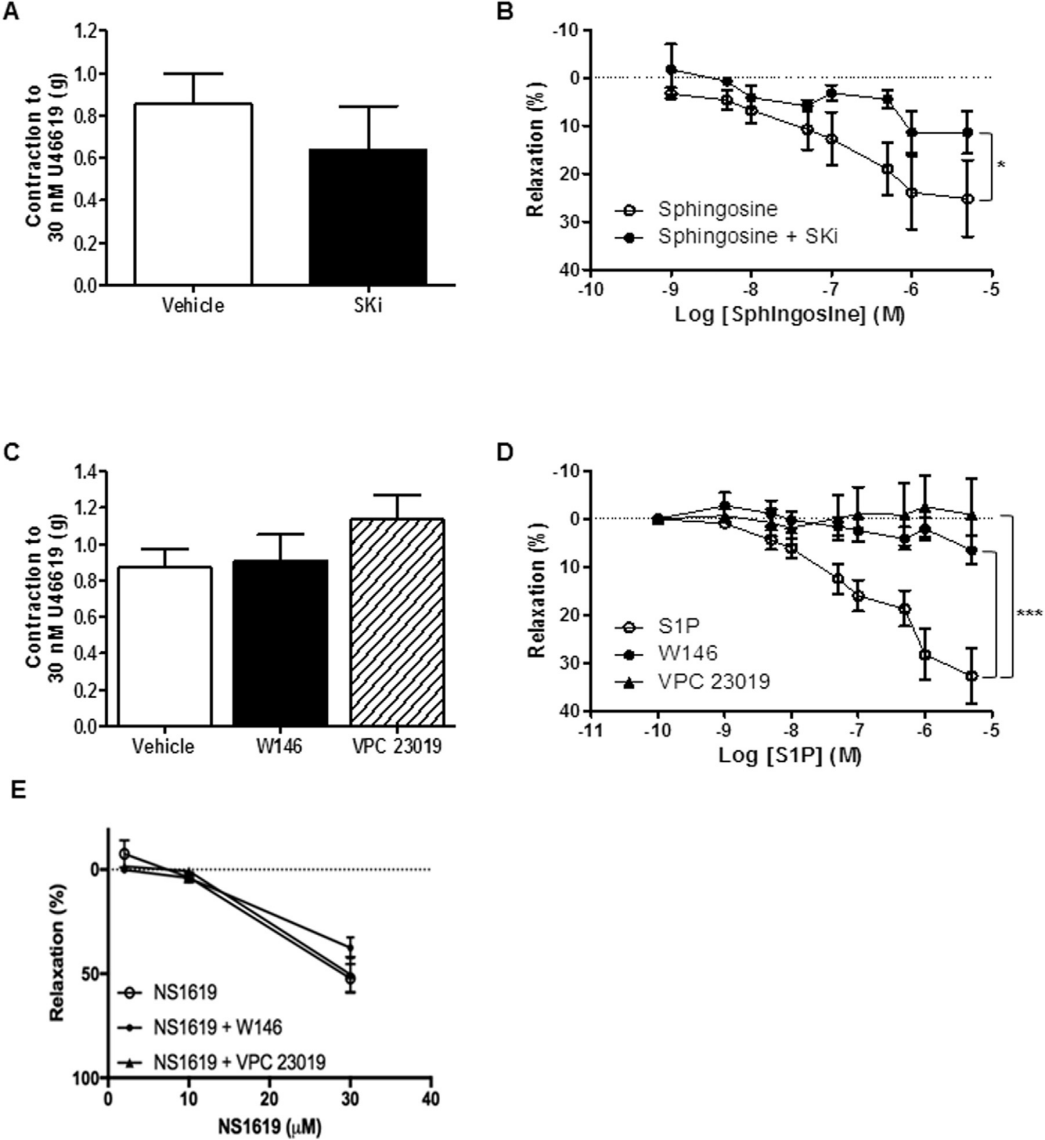
Responses to sphingosine and S1P in the presence and absence of a SK inhibitor and S1P receptor antagonists in denuded mouse aortic rings. (A) Vessels were pre-treated with the dual SK1/2 inhibitor SKi prior to U46619-induced contraction. n = 3–9. (B) Dose-response to sphingosine and in the presence of SKi (10 μM) were produced. *P < 0.05 *versus* sphingosine alone, n = 3–9, two-way ANOVA. (C) Vessels were pre-treated with selective S1P_1_ antagonist W146 or the S1P_1/3_ antagonist, VPC 23019 (both 10 μM) prior to U46619-induced contraction. n = 5–9. (D) Dose-response to S1P in the presence and absence of W146 and VPC 23019 were produced. (E) The BK_Ca_ channel opener NS1619 produced a vasodilation in endothelium-intact aortic rings which was not affected by either W146 or VPC 23019. ***P < 0.001 *versus* S1P alone, n = 5–9, two-way ANOVA.

In denuded, pre-contracted mouse aortic rings, addition of sphingosine or S1P (1 nM to 5 µM) prepared in 4 mg/ml BSA solution (Rosen et al., 2009) induced a dose-dependent relaxation (Fig. 4). SKi had no significant effect on the pre-contraction to U46619 (Fig. 4A). In vessels incubated with SKi prior to pre-contraction with U46619, the relaxation to sphingosine was markedly attenuated (maximum relaxation of 11.4 ± 4.4% with SKi, n = 3 *versus* 25.2 ± 8.0% with vehicle, n = 9, Fig. 4B). In endothelium intact, pre-contracted rings, addition of S1P did not induce any relaxation (n = 5; data not shown) and sphingosine only induced a very small relaxation (maximum of 6.8 ± 2.8%; n = 6) which was not sensitive to SKi (maximum relaxation 7.6 ± 2.9%; n = 5). Similarly, AEA only induced a small relaxation at a concentration of 100 µM (5.9 ± 4.8% (n = 5)) and produced no relaxation in denuded rings (n = 5; data not shown).

In order to determine which S1P receptors on the vasculature are important in the S1P-mediated vasodilation in denuded mouse aortae, vessels were pretreated with either W146 (a S1P_1_ antagonist) or VPC 23019 (a S1P_1/3_ antagonist). Neither W146 nor VPC 23019 affected the preconstriction of the aorta in response to U46619 (Fig. 4C). S1P induced a maximum relaxation of 32.7 ± 5.6% (Fig. 4D) which was almost completely abolished by W146 (maximum relaxation reduced to 6.5 ± 3.1%; n = 8) or VPC 23019 (maximum relaxation was reduced to -1.0 ± 7.6%; n = 5). This suggests that S1P_1_ is the receptor subtype responsible for the vasodilator effect of S1P at the level of vascular smooth muscle cells (VSMCs). To exclude the possibility that VPC23019 and W146 were inhibiting relaxation to S1P by acting as direct BK_Ca_ inhibitors, relaxation to the BK_Ca_ activator NS1619 was studied. In endothelium-intact rings, NS1619 induced a relaxation at 30 µM which was unaffected by pretreatment with either 10 µM VPC 23019 or 10 µM W146 (Fig. 4E).

### 3.4 Distribution of S1P_1_ on the mouse aorta

**Fig. 5:**
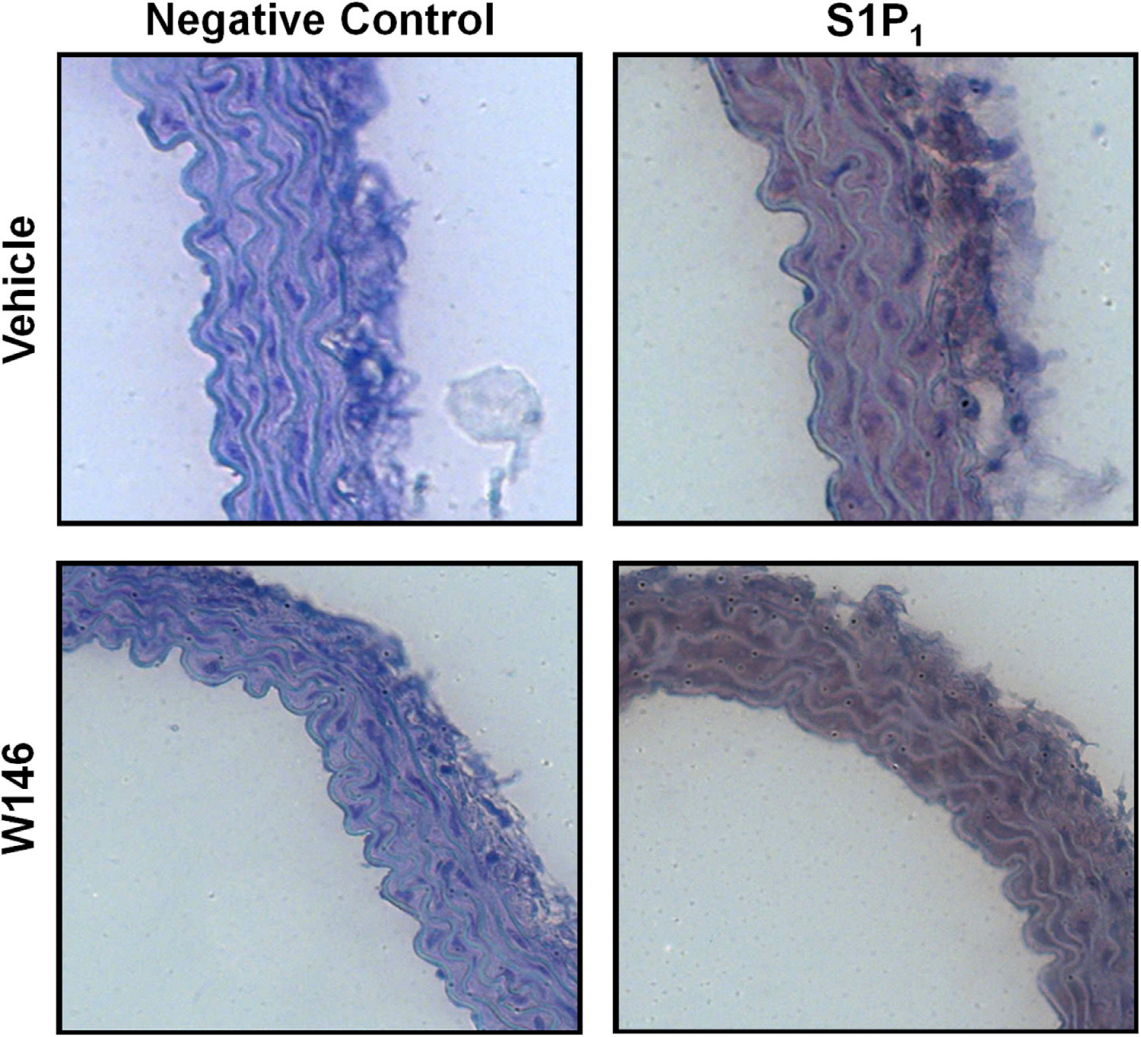
S1P_1_ immunostaining in the endothelium and medial vascular smooth muscle cells of aortae from vehicle- and W146-treated mice. Representative photomicrographs (n = 6) of sections stained with S1P_1_ and counterstained with haematoxylin. Specific staining is seen as a brown colour and was visualised *via* a peroxidase-DAB method (magnification × 400 for all panels).

In human tissue, although S1P_1_ is abundantly expressed on cardiomyocytes and endothelial cells of coronary vessels, it was not found on the aortic smooth muscle, where S1P_2_ and S1P_3_ were expressed (Mazurais et al., 2002). Since our data suggest that S1P_1_ is responsible for S1P-mediated dilation in denuded mouse aortae, we examined S1P_1_ expression on the smooth muscle and endothelium by immunohistochemistry. Expression of S1P_1_ was found throughout the media of the vessels with strong immunostaining in the endothelium. Pretreatment of the mice with W146 did not affect receptor expression pattern or density (Fig. 5).

## 4 Discussion

In this study, we have described a novel mechanism which underlies the phase I hypotensive response to the endocannabinoid AEA in the anaesthetised mouse. The response appears to be dependent on SK1 activity but not SK2 activity, and at least part of the hypotensive response is *via* activation of S1P_1_, presumably through generation and release of S1P. The *in vitro* data indicate that while S1P has a vasodilator effect *via* activation of S1P_1_, this is at the level of the medial smooth muscle rather than the endothelium. Thus, the AEA-induced activation of SK and generation of S1P may occur within resistance vessels but does not occur within conduit vessels such as the aorta.

### 4.1 Effect of SK inhibition on basal MAP

Both BML-258 and ROMe induced a lowering of basal MAP when injected 24 h before measurement. This was particularly marked with the selective SK1 inhibitor BML-258, where the reduction was around 40%. This is in contrast to SK1 knockout mice which present with no change in MAP (Olivera et al., 2010), although this may be due to some compensatory mechanism. Similarly, SKi had no effect on MAP despite lowering basal HR and this may represent an off-target effect of SKi, The lowering of basal MAP strongly suggests that the SK1/S1P-axis participates in the physiological regulation of BP. However, since W146 did not induce any change in basal MAP, it seems that either S1P_1_ is not involved in maintenance of basal MAP or other S1P receptor subtypes can be activated to maintain BP when S1P_1_ is blocked. Other studies (Spijkers et al., 2012) have similarly observed a BP reduction in normotensive rats treated with FTY720 or *N*-*N*-dimethylsphingosine, both of which are SK1 inhibitors (Tonelli et al., 2010). The reduction in basal MAP could be due to effects on TPR or cardiac output (CO) or a combination of both. Basal HR was unchanged by SK inhibition but negative inotropic effects could account for a lowering of CO. However, most studies have claimed that S1P has negative inotropic and chronotropic effects (Landeen et al., 2008, Means et al., 2008); therefore, blocking generation of S1P *in vivo* would be expected to raise CO rather than lowering it. A reduction in TPR in response to blockade of SK is more likely. Indeed, several studies have indicated that an action of S1P on S1P_2_ can cause vasoconstriction of resistance vessels (Bischoff et al., 2000b, Bischoff et al., 2000a) and mice lacking S1P_2_ exhibit an overall reduction in vascular tone and contractile responsiveness (Lorenz et al., 2007). Expression of dominant negative SK1 in VSMCs and resistance arteries decreases vascular tone and myogenic responsiveness (Bolz et al., 2003), all of which could result in a lowering of basal MAP.

### 4.2 SK in anandamide-induced hypotension

Both 1 and 10 mg/kg AEA dose-dependently induced a rapid fall in MAP and HR which was of a similar magnitude to that reported previously (Lake et al., 1997, Malinowska et al., 2001). In a small number of experiments the stable anandamide analogue methanandamide also produced a rapid fall in MAP although this was of a smaller magnitude, suggesting that arachidonic acid metabolites of AEA may play some part in the phase I hypotensive response. Here, we focussed on the phase I hypotensive response to AEA and found it to be dependent on SK1 but not SK2 activity. Indeed, previous studies have noted that cannabinoids can modulate sphingolipid metabolism (Galve-Roperh et al., 2000, Gustafsson et al., 2009, Mair et al., 2010). Moreover, SK1 has been shown to be involved in a number of agonist-induced responses involving S1P generation that subsequently acts *via* so called ‘inside-out’ signalling (Takabe et al., 2008).

It is possible that AEA employs a signalling pathway that induces phosphorylation and activation of SK1 *via* extracellular signal-regulated kinases-1/2, and we previously demonstrated this effect in isolated rat coronary arteries *in vitro* (Mair et al., 2010). Our data do not rule out activation of TRPV1 in response to injection of AEA. Indeed, generation of S1P *via* SK1 may activate TRPV1 to produce bradycardia and also activate S1P_1_ on vessels to produce vasodilation- both of which would contribute to the phase I hypotension. In support of this, the bradycardic effect in response to 10 mg/kg AEA tended to be lower in animals treated with BML-258 (Supplementary Fig. 2) and a recent study has claimed that S1P can sensitise TRPV1 channels on sensory neurons to stimuli (Langeslag et al., 2014). Activation of CB_2_ receptors by AEA may also be involved, based on our own data in the rat (Mair et al., 2010) and an *in vivo* study where injection of the CB_2_ receptor agonist HU-308 induced hypotension (Hanus et al., 1999). An indirect effect of SK1 is also a possibility since sphingosine can act as a CB_1_ antagonist and may blunt the AEA response in animals pretreated with an SK1 inhibitor, where sphingosine is likely to accumulate (Paugh et al., 2006). In the mouse aorta, our *in vitro* data indicate that conversion of sphingosine to S1P is required for vasodilatation to occur and that sphingosine itself does not mediate a vasodilator effect. Blocking this conversion *in vivo* using BML-258 or SKi would prevent the vasodilator actions of endogenous S1P. Interestingly, the presence of the endothelium prevented relaxation to S1P albeit that this was in a conduit vessel. Further experiments are required to assess the vasodilator effects of S1P in an endothelium-intact resistance vessel which would be more relevant to modulating total peripheral resistance.

### 4.3 Effect of S1P_1_ antagonist on the response to anandamide

The S1P_1_ antagonist W146, at a dose used in previous studies (Oo et al., 2011, Tarrason et al., 2011), had a tendency to inhibit the hypotensive response to AEA. However, what was striking was the effect of W146 pretreatment on the change in systolic BP in response to AEA injection. The fall in systolic BP was inhibited by W146 but the fall in diastolic BP in response to AEA was preserved. This will lead to a rise in pulse pressure which is determined by a number of factors including the stroke volume of the heart, compliance of the aorta and the resistance to flow in the arterial tree. S1P_1_ is the predominant isoform expressed on cardiac myocytes (Means and Brown, 2009) and activation causes a negative inotropic effect *via* G_i_ and decreased cAMP within the cardiomyocytes. Consequently, blocking S1P_1_ may lead to an increase in the force of contraction of the heart and an increased pulse pressure following AEA administration. What causes this effect remains to be determined but it is unlikely to be *via* an action of AEA at CB receptors since these are Gi linked and would therefore mediate negative inotropy and chronotropy. In the absence of W146, S1P generated by injection of AEA may blunt the reflex rise in stroke volume, leading to a fall in measured systolic BP. However, W146 may also affect vascular compliance which would increase the pulse pressure and, as we have demonstrated *in vitro* where preincubating aortic rings with W146 almost completely blocked the relaxation in response to S1P. Thus if AEA is causing hypotension through generation of S1P, then inhibition of S1P_1_ in the aorta (and perhaps also resistance arterioles) may ultimately lead to decreased relaxation and an increase in pulse pressure.

### 4.4 Vascular actions of sphingosine and S1P

In this study, S1P and sphingosine both elicited a modest vasodilator response in denuded mouse aortic rings. However, very little relaxation was observed in aortic rings with intact endothelium which is in contrast to the results presented by others (Roviezzo et al., 2014). To our knowledge, this is the first study to characterise the vascular actions of sphingosine in mouse aorta. Our results agree with the effects in porcine aorta (Hsiao et al., 2005) and coronary arteries (Murohara et al., 1996) but are in contrast to studies reported in rat aortae and mesenteric arteries where there was no effect, and in rat renal arteries where there was a vasoconstriction (Johns et al., 1999, Bischoff et al., 2000b). Sphingosine may inhibit Ca^2+^ flux from the sarcoplasmic reticulum and L-type Ca^2+^ channels as observed in cardiomyocytes (McDonough et al., 1994) or it may inhibit protein kinase C, which is activated by U46619 and has contraction-promoting effects (Studer et al., 1994). However, since we found that the dual SK1/2 inhibitor, SKi prevented the relaxation to sphingosine, we conclude that metabolism of sphingosine to S1P and a direct relaxation of VSMCs is the likely mechanism in denuded mouse aortae. S1P is generally reported to constrict denuded vessels (Bischoff et al., 2000b) and to dilate only endothelium-intact mouse and rat thoracic aorta (Nofer et al., 2004, Roviezzo et al., 2006). However, we found S1P to relax denuded aortic rings and this was of a similar magnitude to what we observed in denuded rat coronary artery (Mair et al., 2010).

VSMC abundantly express S1P_2_ and S1P_3_ (Means and Brown, 2009), both of which have been linked to increases in intracellular Ca^2+^ and vasoconstriction, making it unlikely that S1P induces relaxation through these receptors. We speculated that S1P_1_ may be the receptor responsible for S1P-mediated vasodilation. Preincubation of vessels with W146 significantly inhibited the relaxation to S1P, suggesting that S1P_1_ is indeed the main subtype responsible for S1P-induced vasodilation in this vessel. Use of the combined S1P_1/3_ antagonist did not have a significantly greater effect than W146, suggesting that S1P_3_ did not contribute significantly to the response in the mouse aorta. Curiously, in endothelium-intact vessels, the vasodilator response to S1P was not seen, despite S1P_1_ being detected immunohistochemically on the endothelium. It is possible that in a conduit vessel, such as the aorta, the endothelial S1P_1_ receptors are less sensitive or that another S1P receptor subtype opposes the vasodilation mediated *via* activation of S1P_1_ in the VSMCs.

Other studies in resistance vessels rather than conduit vessels have also reported expression of S1P_1_ receptors. In the rat, Yin et al. (2012), demonstrated expression in cardiac microvasculature while a study by Hemmings et al. (2004) used Western blotting of homogenised mesentery and showed expression of S1P_1_ receptor which was decreased in aged rats. In human mesenteric and skeletal muscle resistance vessels, expression of S1P_1–3_ receptors was detected (Hui et al., 2015). In that study the authors noted wide variability in receptor expression between vascular beds (and between species when they studied expression in mouse cremaster skeletal muscle arteries) and so it seems probable that a balance exists between dilator and constrictor effects of S1P dependent on receptor distribution, abundance and access of S1P to these receptors.

### 4.5 Conclusions

In conclusion, this study provides evidence that the SK1/S1P-axis is important not only in physiological BP regulation but is necessary for the phase I hypotension in response to AEA. Generation of S1P in response to AEA likely activates S1P_1_ to reduce TPR and lower MAP. In conduit vessels, this is at the level of the VSMCs while in resistance vessels, an effect of S1P receptors on the endothelium may also contribute, although this remains to be determined. These findings have important implications in our understanding of the hypotensive and cardioprotective actions of cannabinoids and may aid the identification of novel pharmacological targets for the treatment of hypertension.

## Funding

This work was supported by the Integrative Mammalian Biology Initiative, jointly funded by the BBSRC and MRC and by a British Heart Foundation Ph.D. Studentship (FS/08/071/26212) to FHG.

## Conflicts of interest

None of the authors has any conflict of interest to declare.

## List of author contributions

FH Greig, K Nather, MD Ballantyne, Z Kazi, H Alganga, MA Ewart, KE Zaborska & S Kennedy all performed experiments, gathered data and undertook statistical analysis of the data.

S Kennedy wrote the first draft of the manuscript.

NJ Pyne, S Pyne & S Kennedy rewrote parts of the manuscript and finalised submission.
